# Innovative Therapeutic Strategy for Complicated Thoracoabdominal Aortic Aneurysms: Dimension Reduction

**DOI:** 10.1016/j.jaccas.2025.103904

**Published:** 2025-05-14

**Authors:** Shuangjing Wang, Weiguo Fu, Lixin Wang

**Affiliations:** a Department of Vascular Surgery, Zhongshan Hospital, Fudan University, Shanghai, China; b Institute of Vascular Surgery, Zhongshan Hospital, Fudan University, Shanghai, China; c National Clinical Research Center for Interventional Medicine, Shanghai, China

**Keywords:** dimension reduction, open surgical repair, staged hybrid repair, thoracoabdominal aortic aneurysms

## Abstract

Open surgical repair of complicated thoracoabdominal aortic aneurysms (TAAAs) has a high mortality rate, leading to an increased appreciation for staged hybrid repair. We report a case of abdominal aneurysm replacement with an artificial Y graft and total visceral aortic debranching through retrograde artery revascularization with a customized 3-bifurcated graft in a hybrid surgery for type II TAAA. Staged hybrid repair epitomizes the application of a dimensionality reduction treatment concept, offering considerable promise for addressing complicated TAAAs.

## Graphical Abstract

**Figure:**
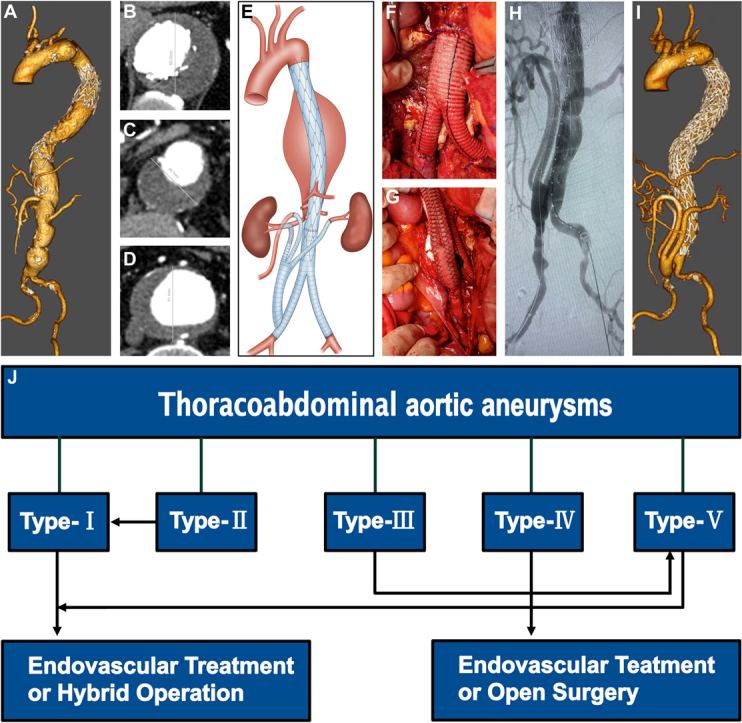

A 68-year-old man with a type II thoracoabdominal aortic aneurysm (TAAA) underwent thoracic endovascular aortic repair at another hospital 2 months previously (Figure 1A). He was referred to us for further management (Figures 1B to 1D). Open surgical repair has been the gold standard for treating TAAAs for decades, but it has been challenged by high mortality rates and the favorable outcomes of endovascular approaches.^1^ In addition, open surgical repair has been relegated to younger and fit patients with genetic syndromes and those who are not suitable anatomic candidates for endovascular repair.^2^ A hybrid treatment plan was designed for this patient. In this case, the treatment protocol can be categorized as a staged hybrid repair (Figure 1E). During hybrid surgery, the abdominal aneurysm was resected and replaced with an artificial customized Y graft. The customized artificial Y graft was anastomosed end-to-end with the abdominal aorta and the 2 common iliac arteries (Figure 1F). Subsequently, a side-to-end anastomosis was performed between the distal end of the right iliac artery prosthesis and a customized 3-bifurcated graft (Figure 1G). Two branches were separately anastomosed in an end-to-end manner to the superior mesenteric artery and the left renal artery, respectively. A surgical fenestration was created in the polytetrafluoroethylene graft, through which a GORE VIABAHN stent was delivered precisely under fluoroscopic guidance, achieving a 3-cm overlap with the native right renal artery lumen and a 5-cm overlap with the prosthetic graft segment. A completion angiogram demonstrated that the TAAA was completely repaired, and the superior mesenteric artery and bilateral renal arteries were revascularized (Figure 1H). Hemostasis was achieved, the graft was wrapped with the aneurysm wall, and the retroperitoneum was closed. The patient was then transferred to the intensive care unit.

**Figure 1:**
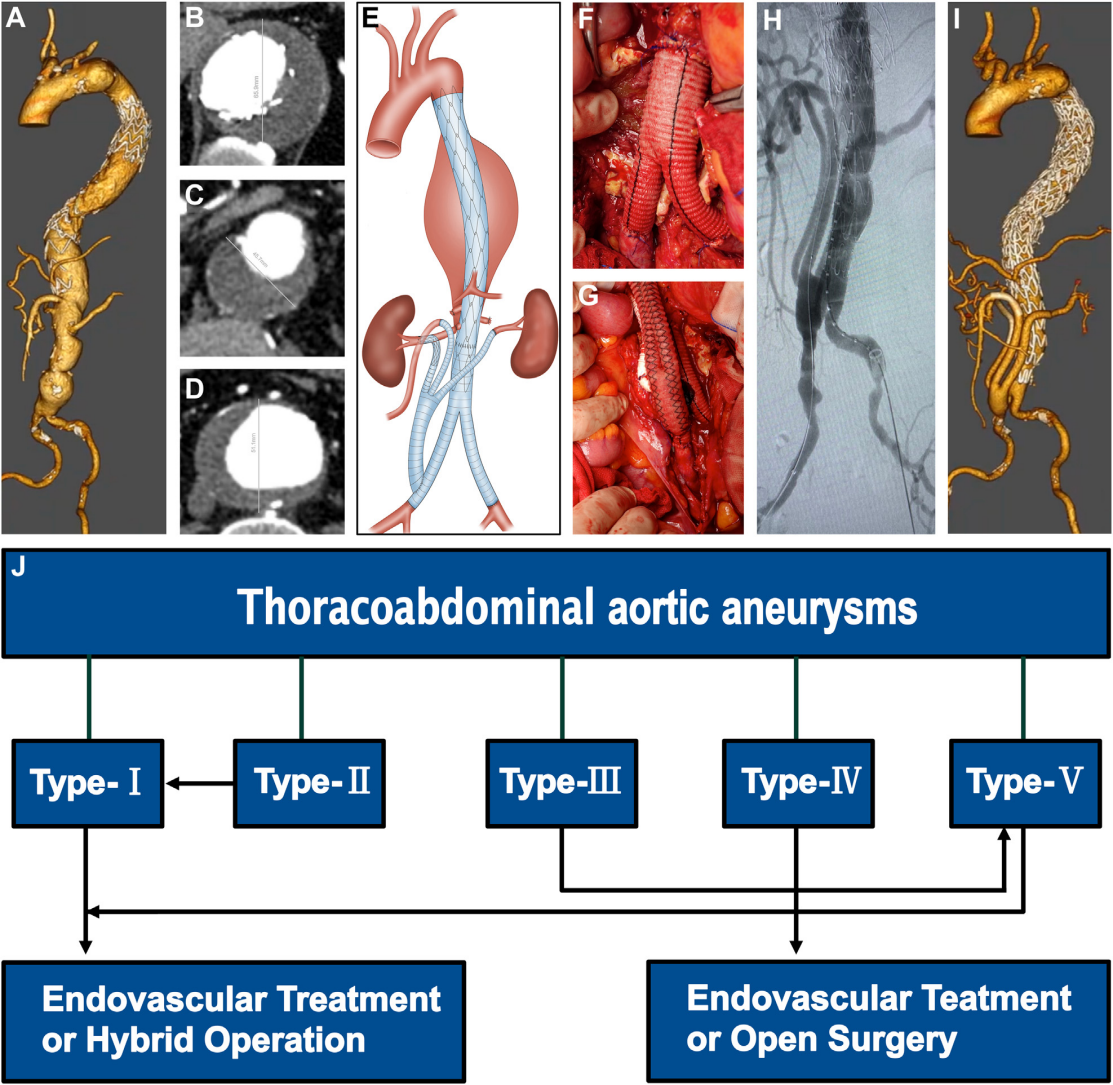
Dimension Reduction in TAAA (A) 3-dimensional CTA (3D-CTA) revealing multiple aortic aneurysms and (B to D) measurements of the diameters with the automatic 3-dimensional sizing software Endosize (Therenva). (E) A 2-stage hybrid strategy for this patient. (F) A customized artificial Y-graft was anastomosed in an end-to-end fashion to the abdominal aorta and common iliac arteries. (G) A customized trifurcated graft was anastomosed in a side-to-end fashion to the distal portion of the right iliac arterial prosthesis. (H) Completion angiogram demonstrated that TAAA was completely repaired, and the superior mesenteric artery and bilateral renal arteries were revascularized. (I) Follow-up CTA demonstrating satisfactory TAAA repair, revascularization of superior mesenteric artery and bilateral renal arteries, and no evidence of endoleak. (J) Treatment flow chart for TAAA. CTA = computed tomography angiography; TAAA = thoracoabdominal aortic aneurysm.

After a satisfactory clinical and laboratory assessment, the patient was discharged. He was followed up 3 months later at the clinic. Computed tomography angiography during this visit revealed satisfactory TAAA repair, revascularization of the superior mesenteric artery and bilateral renal arteries, and no evidence of endoleak (Figure 1I).

We define the process of reducing a type II TAAA to a type I TAAA after removal of the abdominal aneurysm as “dimension reduction.” This case highlights that dimension reduction is an innovative therapeutic strategy for complicated TAAAs. Consequently, we propose that staged hybrid repair should be applied to a type III TAAA, transforming it into type V, which facilitates spatial dimensionality reduction and improves patient outcomes. This case contributes to the evolving landscape of aortic aneurysm interventions. Further studies are still needed to demonstrate the safety and efficacy of the dimension reduction therapeutic strategy.

## Funding Support and Author Disclosures

Supported in part by the National Natural Science Foundation of China (grant no. 82270415). The authors have reported that they have no relationships relevant to the contents of this paper to disclose.
